# Comparative Evaluation of *Chlorella vulgaris* and *Anabaena variabilis* for Phycoremediation of Polluted River Water: Spotlighting Heavy Metals Detoxification

**DOI:** 10.3390/biology12050675

**Published:** 2023-05-01

**Authors:** Md. Shakir Ahammed, Md. Abdul Baten, Muhammad Aslam Ali, Shahin Mahmud, Md. Sirajul Islam, Bhim Sen Thapa, Md. Aminul Islam, Md. Alim Miah, Tanmoy Roy Tusher

**Affiliations:** 1 Department of Environmental Science, Bangladesh Agricultural University, Mymensingh 2202, Bangladesh; shakiresrm@gmail.com (M.S.A.); baten.envsc@bau.edu.bd (M.A.B.); aminul.aim.25@gmail.com (M.A.I.); 2 Department of Biotechnology and Genetic Engineering, Mawlana Bhashani Science and Technology University, Tangail 1902, Bangladesh; shahin018mbstu@gmail.com; 3 Department of Environmental Science and Resource Management, Mawlana Bhashani Science and Technology University, Tangail 1902, Bangladesh; islammstazu@yahoo.com; 4 Department of Biological Sciences, Marquette University, Milwaukee, WI 53233, USA; bhimsen.thapa@marquette.edu; 5 Department of Environmental Science and Engineering, Jatiya Kabi Kazi Nazrul Islam University, Trishal, Mymensingh 2224, Bangladesh; alim@jkkniu.edu.bd

**Keywords:** bioremediation, microalgae, cyanobacteria, heavy metals, pollutant removal

## Abstract

**Simple Summary:**

Globally, rivers are continuously being polluted because of anthropogenic discharge, especially in Asian countries experiencing rapid urban, industrial and agricultural developments. Exceedingly high concentrations of nutrients and toxic metals have been detected in most Asian rivers, which has led to major environmental and human health concerns that demand the detoxification of polluted river water. This study investigated and compared the efficacy of microalgae (*Chlorella vulgaris*) and cyanobacteria (*Anabaena variabilis*) as a low-cost and eco-friendly approach to remediate polluted river water. The results revealed that both microalgae and cyanobacteria have the potential to reduce the pollutant load from the raw river water, but the removal efficiency is species dependent. The studied microalgal and cyanobacterial species are excellent candidates for polluted water and/or wastewater treatment as well as producers of energy-rich biomass that can be further processed to produce biofuel, biodiesel, and other bio-hydrocarbons.

**Abstract:**

This study investigated the phycoremediation abilities of *Chlorella vulgaris* (microalga) and *Anabaena variabilis* (cyanobacterium) for the detoxification of polluted river water. Lab-scale phycoremediation experiments were conducted for 20 days at 30 °C using the microalgal and cyanobacterial strains and water samples collected from the Dhaleswari river in Bangladesh. The physicochemical properties such as electrical conductivity (EC), total dissolved solids (TDS), biological oxygen demand (BOD), hardness ions, and heavy metals of the collected water samples indicated that the river water is highly polluted. The results of the phycoremediation experiments demonstrated that both microalgal and cyanobacterial species significantly reduced the pollutant load and heavy metal concentrations of the river water. The pH of the river water was significantly raised from 6.97 to 8.07 and 8.28 by *C. vulgaris* and *A. variabilis*, respectively. *A. variabilis* demonstrated higher efficacy than *C. vulgaris* in reducing the EC, TDS, and BOD of the polluted river water and was more effective at reducing the pollutant load of SO_4_^2−^ and Zn. In regard to hardness ions and heavy metal detoxification, *C. vulgaris* performed better at removing Ca^2+^, Mg^2+^, Cr, and Mn. These findings indicate that both microalgae and cyanobacteria have great potential to remove various pollutants, especially heavy metals, from the polluted river water as part of a low-cost, easily controllable, environmentally friendly remediation strategy. Nevertheless, the composition of polluted water should be assessed prior to the designing of microalgae- or cyanobacteria-based remediation technology, since the pollutant removal efficiency is found to be species dependent.

## 1. Introduction

Water is the primary requirement for the survival of all life forms [1], and when it comes to the human utilization of it, water quality is significantly more essential than water quantity [2]. Rivers are an important natural source of water primarily for domestic, industrial, and agricultural uses as well as being a vital habitat for numerous freshwater organisms [3,4]. However, rivers around the world are being severely polluted due to increased human activities such as the release of wastewaters from industrial, commercial, municipal, domestic, and agricultural sources [5,6]. All these wastewaters are known to carry a wide variety of inorganic and organic pollutants in addition to heavy metals such as chromium (Cr), manganese (Mn), lead (Pb), zinc (Zn), copper (Cu), etc. [6,7]. The presence of heavy metals in river water has become a serious environmental and public health concern because of its toxicity, carcinogenicity, mutagenicity and teratogenicity even at low concentrations, non-biodegradability, persistence in the aquatic environment, and potential for bioaccumulation and biomagnifications in aquatic organisms [6,8,9]. In addition to toxic heavy metals, the excessive and repeated discharge of wastewaters into rivers may substantially increase the levels of electrical conductivity (EC), total dissolved solids (TDS), total suspended solids (TSS), nutrients (e.g., nitrate (NO_3_^−^), sulfate (SO_4_^2−^), and phosphate (PO_4_^3−^)), total hardness (Ca^2+^ and Mg^2+^), total alkalinity, chemical oxygen demand (COD), and biological oxygen demand (BOD) in the water, resulting in the degradation of water quality and adverse effects on the aquatic organisms [10,11]. Since the wastewaters have distinct physicochemical properties that are mixed together in the rivers [12], the properties of the river water end up being completely changed and challenging to treat.

Previous methods proven to be effective in treating polluted river water have included sedimentation, flocculation, coagulation, precipitation, oxidation, ion exchange, membrane filtration, and electrocoagulation [4,6]. However, these physicochemical treatments are usually discouraged due to high operation and maintenance costs, energy requirement, and possible secondary contamination [13,14]. Phycoremediation is considered to be a more cost-effective, energy-efficient, eco-friendly bioremediation option compared to the conventional physicochemical treatments. Phycoremediation employs microalgae and/or cyanobacteria to clean up water and/or wastewater by removing heavy metals, eliminating excess nutrients, and successfully fixing carbon dioxide (CO_2_) through photosynthesis [15,16,17]. Because of their simple structure and high photosynthetic efficiency, both eukaryotic microalgae and prokaryotic cyanobacteria can survive and grow in adverse environmental conditions including extreme temperature, high salinity, a wide range of pH levels, nutritional stress, and the presence of wastewater toxins [6,16,18]. Additionally, the removal of carbon and other nutrients from wastewater by microalgae and/or cyanobacteria can increase biomass production, which can then be converted into high-value bioproducts and biofuels [19,20].

A plethora of microalgal genera (e.g., *Botryococcus*, *Chlamydomonas*, *Chlorella*, *Chlorococcum*, *Gloeocystis*, *Scenedesmus*, etc.) and cyanobacterial genera (e.g., *Anabaena*, *Chroococcus*, *Limnothrix*, *Limnospira*, *Nostoc*, *Phormidium*, *Planktothrix*, etc.) have been reported to be effective at detoxifying polluted water and wastewater [4,13,21,22,23,24]. Microalgae have been substantially employed for the remediation of heavy metals due to their large surface area, high binding affinity, and high abundance of binding sites [25]. On the other hand, the presence of polysaccharides (i.e., extracellular polymeric substances or EPS) and diverse proteins on the cyanobacterial surface also provides an enormous number of binding sites for the heavy metals [26]. Among the microalgal genera, *Chlamydomonas*, *Chlorella*, and *Scenedesmus* have been extensively used for phycoremediation studies [23,27], while the species belonging to the cyanobacterial genera *Anabaena* and *Nostoc* have merely been utilized and studied [26]. However, most of these microalgae- and cyanobacteria-based phycoremediation studies primarily focused on the removal of nutrients and/or pollutants from a particular industrial wastewater such as textile, tannery, poultry, or dairy wastewater [28]. Only a few studies are available in the literature which evaluated the phycoremediation potential of microalgal and/or cyanobacterial species for polluted river or lake water [4,19,28,29], while Koul et al. [28] barely studied and compared the Pb(II) remediation potential. Therefore, more studies are needed to be conducted to broaden our knowledge and understanding about the phycoremediation potential of diverse microalgal and cyanobacterial species.

The rivers of Bangladesh are frequently reported for their worst water quality, heavy metal pollution, and risk to ecological and human health [3,30]. Based on the existing literature, no study is available on the microalgae- or cyanobacteria-assisted phycoremediation of Bangladeshi river water. This study, for the first time, aims to evaluate and compare the efficacy of *Chlorella vulgaris* as a microalga and *Anabaena variabilis* as a cyanobacterium for the phycoremediation of polluted river water of Bangladesh. Moreover, since the growth and metabolic activities of both microalgae and cyanobacteria essentially vary depending on the wastewater compositions, such comparative studies are of paramount importance to expand our knowledge about the efficacy of microalgal and cyanobacterial species that have the distinct characteristics needed to be used in wastewater-specific phycoremediation technologies [17,29]. In this study, Dhaleswari river water was selected because of the recent study reporting that the Dhaleswari river water is severely polluted with high organic load, TDS, and toxic heavy metals [31]. The objectives of this study were: (i) to investigate the potential of *C. vulgaris* and *A. variabilis* in improving the physicochemical parameters of the river water (pH, EC, TDS, BOD, Mg^2+^, Ca^2+^, and SO_4_^2−^) as well as removing the heavy metals (Zn, Cr, and Mn) from the water, and (ii) to assess the growth of studied microalga and cyanobacterium in the polluted river water.

## 2. Materials and Methods

### 2.1. Sampling and Analysis of River Water

River water samples were collected from five different locations along the Dhaleswari river in Bangladesh. Before sample collection, the sampling bottles were cleaned, washed, and treated with 5% nitric acid (HNO_3_) overnight. The bottles were then dried and washed with deionized water. During collection, the pre-prepared sampling bottles were submerged about 10 cm beneath the water surface [32]. A composite water sample was made by mixing the water samples collected from three sampling points at each sampling location. The pH, EC, dissolved oxygen (DO), and TDS of the composite sample were immediately analyzed using respective digital meters. The sampling bottles were labeled with the corresponding identification number, and a few drops of diluted HNO_3_ was immediately added to the water samples to avoid the elemental loss [4]. All five bottles were tightly screwed and transported to the laboratory for further analysis with care taken to avoid exposing the samples to sunlight and temperature changes.

The water quality parameters such as pH were determined by the digital pH meter (pH Scan WP, Eutech Instruments, Selangor, Malaysia). A digital EC and TDS meter (HM digital, Inc., Culver city, CA, USA) were used to determine EC and TDS, respectively. The DO was determined by a digital DO meter (Lutron Electronic Co., Ltd., Taipei, Taiwan) using sodium thiosulfate (0.025 N). The BOD (DO_0_-DO_5_; where, DO_0_ = Initial DO in the sample, and DO_5_ = DO after 5 days) was measured as reported by Huq and Alam [33]. The atomic absorption spectrophotometer (AAS, Shimadzu Corporation, Kyoto, Japan) was used for determining total Zn, Cr, and Mn concentrations in the river water following wet oxidation of the samples by the di-acid digestion method with a mixture (3:1) of concentrated HNO_3_ and perchloric acid (HClO_4_) [34]. The mean values of the observed physicochemical properties of the collected composite river water samples were considered as the day 0 parameters for phycoremediation experiments.

### 2.2. Microalgal and Cyanobacterial Species, Culture Medium, and Culturing Conditions

One microalgal species, i.e., *C. vulgaris* and one cyanobacterial species, i.e., *A. variabilis* were used in this study; they were isolated and identified through the morphological (macro and microscopic) observations. Both microalgal and cyanobacterial species were cultured in conical flasks containing BG11 medium and incubated at 30 °C and 190 rpm for 20 days. The improvised BG11 medium (pH = 7.1) contained sodium nitrate (NaNO_3_), 1.5 g; dipotassium hydrogen phosphate (K_2_HPO_4_), 0.04 g; magnesium sulfate (MgSO_4_.7H_2_O), 0.075 g; calcium chloride (CaCl_2_·2H_2_O), 0.36 g; citric acid (C_6_H_8_O_7_), 0.036 g; ammonium ferric citrate ((NH_4_)_5_Fe(C_6_H_4_O_7_)_2_), 0.006 g; EDTA disodium salt, 0.006 g; boric acid (H_3_BO_3_), 2.86 g; manganese chloride (MnCl_2_·4H_2_O), 1.81 g; zinc sulfate (ZnSO_4_·7H_2_O), 0.222 g; sodium molybdate (Na_2_Mo_3_·2H_2_O), 0.39 g; copper sulfate (CuSO_4_·5H_2_O), 0.07 g; and cobalt nitrate (CO(NO_3_)_2_·6H_2_O), 0.07 g [35]. The culturing environment was suitably illuminated to promote the growth and development of tested microalga and cyanobacterium [36].

### 2.3. Phycoremediation Experimental Set Up

Lab-scale phycoremediation experiments were conducted in conical flasks containing 200 mL of autoclaved (at 121 °C for 30 min) composite river water samples to avoid the contamination of indigenous river water microorganisms. For the experiments aiming at BOD removal, unautoclaved water samples were used, as autoclaving may alter the contents of DO and BOD of water. The well-grown *C. vulgaris* and *A. variabilis* cultures were harvested by centrifugation at 6000 rpm for 15 min [4], and the supernatant was discarded. The harvested cells of *C. vulgaris* and *A. variabilis* were then separately inoculated into the corresponding flasks containing river water samples, while the amount of inoculum (0.25 g/L) was maintained same for both species. The inoculum for each species was adjusted by measuring the dry weight biomass based on the total volatile suspended solids (TVSS), as described in Section 2.5. Abiotic controls were also prepared without inoculation, and all the experiments were performed in quintuplicates. The experiments were performed aerobically for 20 days under illumination at 30 °C. The flasks were cultured on a rotary shaker at 100 rpm in the presence of a white fluorescent lamp with an intensity of 120 mol m/s.

### 2.4. Analytical Procedures

Samples were collected every 5 days over the course of a 20-day period, and the physicochemical properties of the river water were analyzed similarly as described in Section 2.1. Prior to the analysis, the samples were filtered through 0.22 µm membrane filters to remove the microalgal or cyanobacterial cells.

### 2.5. Determination of Microalgal and Cyanobacterial Growth

The growth rates of *C. vulgaris* and *A. variabilis* were measured by assessing the TVSS, which reflects the dry weight biomass concentration, using the published method [37]. Briefly, 5 mL of microalgal or cyanobacterial culture was collected and filtered through a 0.22 µm membrane filter (47 mm in diameter), which was dried overnight at 105 °C. The dried sample was then ignited at 550 °C for 30 min. The weight difference between ignited (W_1_) and dried (W_2_) samples was considered as cell biomass (g/L), which is usually proportional to the growth rate of the cells. Based on TVSS, the following equation (Equation (1)) quantifies the microalgal and cyanobacterial growth rates:

RTVSS = ln(TVSSt) − ln(TVSS0)/t(1)

where R shows the growth rate of microalga or cyanobacterium based on TVSS, TVSSt is TVSS at time t and TVSS0 is TVSS at day 0. Time interval (t) represents number of days.

### 2.6. Phycoremediation Efficiency

The phycoremediation efficiencies of *C. vulgaris* and *A. variabilis* for each physicochemical parameters including heavy metals (Zn, Cr, and Mn) on day 20 were calculated by using the following equation (Equation (2)):

Removal efficiency (%) = [(CI − CF) / CI] × 100(2)

where CI and CF are the initial (day 0) and final (day 20) concentrations.

### 2.7. Statistical Analysis

As all the experiments were performed in quintuplicates using the composite river water samples, the values are presented as mean ± standard deviations (SD). The analysis of variance (ANOVA) was performed using IBM SPSS 25.0 to show the significant differences among the treatments’ efficiencies, while the level of significance was set at *p* < 0.05. Linear regression analysis was also performed using Microsoft Excel 2010 to depict the trend of microalgae- and cyanobacteria-assisted phycoremediation of river water samples.

## 3. Results

### 3.1. Physicochemical Properties of Raw River Water

The physicochemical properties of the raw water collected from the Dhaleswari river are presented in Table 1. The pH of the river water was found to be slightly acidic but was within the Bangladesh surface water quality standard (BSWQS) [38]. However, the values of EC (1573.93 µS/cm), TDS (935.55 mg/L), and BOD (17.06 mg/L) exceeded the BSWQS for fisheries and aquatic environment [38,39]. The concentrations of Ca^2+^ and Mg^2+^ together show the total hardness of the river water [40]. In this study, the Ca^2+^ and Mg^2+^ concentrations were found to be 84.04 mg/L and 69.54 mg/L, respectively, indicating a total hardness of 153.58 mg/L that exceeded the recommended value for fisheries or the aquatic environment [33]. The SO_4_^2−^ concentration was measured 117.62 mg/L and found to be within the standard level [2]. As for the heavy metals, the collected river water was heavily contaminated with Cr (0.81 mg/L) and Mn (0.65 mg/L), whereas the concentration of Zn was measured 0.35 mg/L. Considering all of the resulting physicochemical values, the water samples collected from the Dhaleswari river were identified as highly polluted.

### 3.2. Pollutant Removal Efficacy of C. vulgaris and A. variabilis

**Figure 1:**
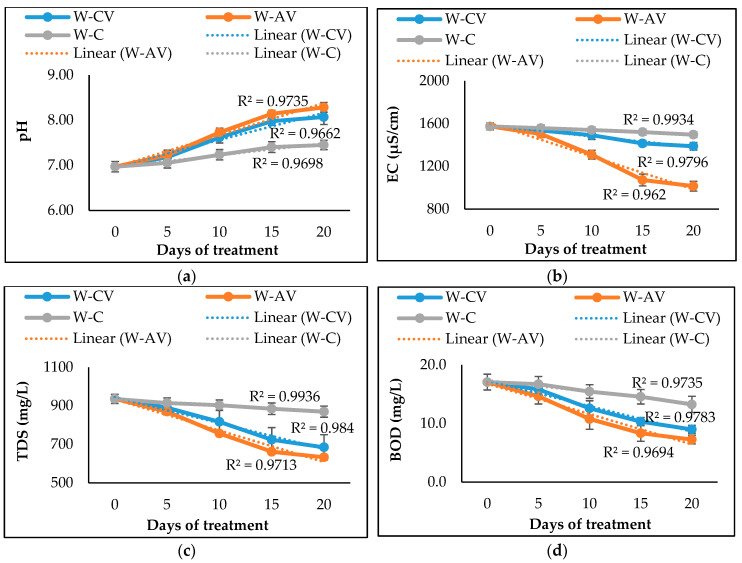
Changes in pH (**a**), EC (**b**), TDS (**c**), and BOD (**d**) of river water after 20 days of treatment by microalgal and cyanobacterial species. Here, W-CV and W-AV represent the water treated by *C. vulgaris* and *A. variabilis*, respectively, and W-C represents the untreated water.

**Figure 2:**
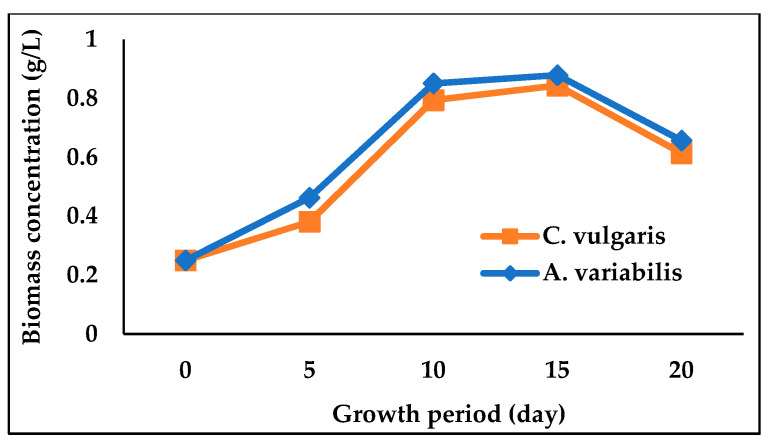
Growth of *C. vulgaris* and *A. variabilis* in raw river water.

The physicochemical properties of the river water after treating for 20 days by *C. vulgaris* and *A. variabilis* are presented in Table 2. The pH of the water was increased after 20 days for both treated and untreated water when compared to the pH of raw water samples; however, the presence of *C. vulgaris* and *A. variabilis* significantly (*p* < 0.05) increased the pH to 8.07 and 8.28, respectively. The addition of microalga or cyanobacterium also substantially reduced the EC, TDS and BOD of the water, with cyanobacterium *A. variabilis* showing a greater reduction than microalga *C. vulgaris* (Table 2). After 20 days of treatment by *A. variabilis*, the EC, TDS, and BOD of the river water were decreased by 35.54%, 32.26%, and 57.56%, respectively. These results indicated that both algal species reduced the pollutant load and subsequently improved the quality of river water. In Figure 1, *C. vulgaris-* and *A.* variabilis-treated water saw a sharp increase in pH level and a decrease in EC, TDS, and BOD levels. Along with the pollutant removal, the growth curves of *C. vulgaris* and *A. variabilis* in river water showed almost identical patterns, although slightly higher growth was observed for *A. variabilis* (Figure 2). The growth curves clearly indicate that both these microalgal and cyanobacterial species exhibited excellent ability to acclimatize and grow in polluted river water.

**Figure 3:**
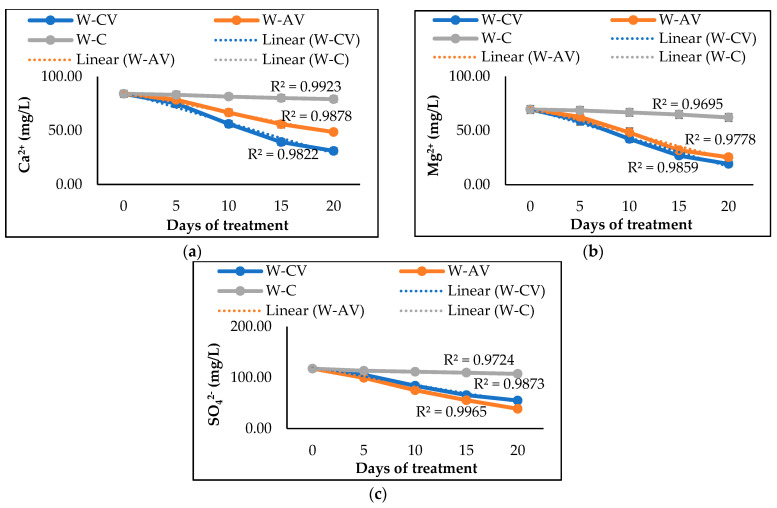
Changes of Ca^2+^ (**a**), Mg^2+^ (**b**), and SO_4_^2−^ (**c**) contents in river water after 20 days of treatment by microalgal and cyanobacterial species. Here, W-CV and W-AV represent the water treated by *C. vulgaris* and *A. variabilis*, respectively, and W-C represents the untreated water.

In addition, the results revealed that *C. vulgaris* and *A. variabilis* have distinct abilities to remove hardness ions from polluted water. *C. vulgaris* showed higher removal efficiency, resulting in 63% Ca^2+^ ion removal and 72% Mg^2+^ ion removal (Table 2). *A. variabilis* was only able to remove 42% of Ca^2+^ ions and 63% of Mg^2+^ ions from the polluted water. However, *A. variabilis* exhibited greater potential to remove SO_4_^2−^ ions, showing 67% removal of SO_4_^2−^ ions, while *C. vulgaris* achieved only 53% removal. Figure 3 shows the trends of Ca^2+^, Mg^2+^, and SO_4_^2−^ ions removal by *C. vulgaris* and *A. variabilis* during 20 days of treatments, which were found to be identical to the removal trends of EC and TDS, as shown in Figure 1. These findings suggest that the studied microalga and cyanobacterium could be applied to reduce the water hardness and pollutant load from the polluted water of environmental relevance.

**Figure 4:**
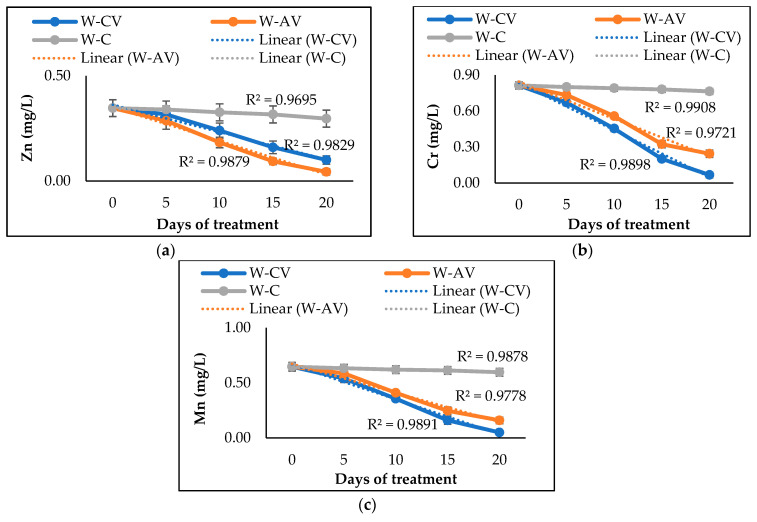
Changes of Zn (**a**), Cr (**b**), and Mn (**c**) concentrations in river water after 20 days of treatment by microalgal and cyanobacterial species. Here, W-CV and W-AV represent the water treated by *C. vulgaris* and *A. variabilis*, respectively, and W-C represents the untreated water.

In case of heavy metals, the studied microalgal and cyanobacterial species also possessed distinct removal abilities. After 20 days, *A. variabilis* demonstrated higher Zn removal efficiency, whereas *C. vulgaris* performed better at removing Cr and Mn (Table 2). The presence of *A. variabilis* and *C. vulgaris* removed ~89% and 71% of Zn from the river water, respectively. In addition, *C. vulgaris* removed 91% Cr and 92% Mn from the river water, resulting in greater efficiency than *A. variabilis* that removed 70% Cr and 75% Mn from the water. Comparing with abiotic controls, the removal of cations and anions was significantly (*p* < 0.05) higher for the microalgal or cyanobacterial treatment (Table 2). Figure 4 presents the heavy metal removal profiles of the studied microalga and cyanobacterium over 20 days of phycoremediation experiments. Both microalgal and cyanobacterial species showed their ability to tolerate and absorb various heavy metals from the polluted water, thus decreasing the overall heavy metal concentrations in the river water.

## 4. Discussion

The microalgae- and/or cyanobacteria-mediated phycoremediation of wastewater is considered a cost-effective and eco-friendly bioremediation method that has been used for over 60 years [42]. Although numerous studies have been performed to assess the possibility of microalgal or cyanobacterial species for wastewater treatment, their potential to be used for the phycoremediation of polluted surface water has rarely been investigated. In this study, the efficiencies of the widely studied *Chlorella* sp. and the less studied *Anabaena* sp. in removing pollutants from raw polluted river water were analyzed and compared to data from previously reported microalgal/cyanobacterial species that were tested on different polluted waters (Table 3).

*C. vulgaris* and *A. variabilis* were substantially able to grow and remediate the polluted water collected from the Dhaleswari river. The growth profiles of *C. vulgaris* and *A. variabilis* were identical to each other in both river water and wastewater environments as observed in this study and other studies by Kumar et al. [45] and Deb et al. [46]. As the experiment progressed, both microalgae- and cyanobacteria-treated samples showed an increase in water pH (Figure 1a), which could be attributed to the photosynthetic CO_2_ assimilation [47]. It has been previously reported that the pH of all types of wastewaters is slightly increased during the microalgal biomass generation due to the algal photosynthetic activity [48]. Zepernick et al. [49] also reported that an increase in water pH can co-occur with the cyanobacterial growth or bloom. *C. vulgaris* and *A. variabilis* also caused a decrease in EC and TDS of the river water (Figure 1b,c), which is possibly due to the utilization of nutrients present in the polluted river water. Peng et al. [50] demonstrated the TDS removal from various wastewaters by an algal biofilm reactor and reported that the removal of EC and TDS is associated with the absorption of ionic elements (of Na, K, Ca, Mg, S, etc.) present in the polluted water.

Our study demonstrated significant removal of Ca^2+^, Mg^2+^, and SO_4_^2−^ from the polluted river water by the studied microalga (*C. vulgaris*) and cyanobacterium (*A. variabilis*), with *C. vulgaris* showing higher Ca^2+^ and Mg^2+^ removal efficiency and *A. variabilis* showing higher SO_4_^2−^ removal efficiency (Table 2). Unlike toxic heavy metals, Ca and Mg are essential for the growth and development of microalgae and cyanobacteria, since both influence the photosynthetic enzymatic activities as well as other enzymes regulating different cell activities [51,52]. Moreover, the increase in the pH of the water also helps to remove the TDS as well as hardness ions [53,54]. A similar association of increase in pH and decrease in EC and TDS is also observed in our study. In addition to hardness ions (Ca^2+^ and Mg^2+^), the studied microalga and cyanobacterium are also found to be capable of removing 53–67% SO_4_^2−^ from the polluted river water, which is considerably higher than the SO_4_^2−^ removal efficiencies of *Chlamydomonas* sp., *Oocystis* sp., *Scenedesmus* sp., and *Fischerella* sp. [55]. Our microalgal and cyanobacterial species also effectively reduced the BOD of the river water, with *A. variabilis* exhibiting greater efficiency by removing ~58% of BOD (Table 2), which is due to the consumption of carbon by microalgal and cyanobacterial species for their growth and development [48]. These results clearly indicate that the pollutant removal efficiencies are essentially species dependent owing to: (i) the differences in their large surface to volume ratios [56], (ii) the differences in the physiological components (e.g., metal-binding groups) that promote metal adsorption, uptake and/or accumulation [56,57], (iii) the differences in the availability of transport systems, storage systems, and catabolic enzyme machinery [56,57], and/or (iv) the differences in ionic strength of different species as well as demand for particular ions [58].

Our microalga and cyanobacterium also exhibited great potential to remove all three tested heavy metals (Zn, Cr, and Mn), although the removal efficiency varies among species due to differing levels of tolerance, survival rates, and removal efficiencies in contaminated waters [59]. In our study, *A. variabilis* was found to be more effective than *C. vulgaris* at removing Zn (~89%) from the river water samples, whereas *C. vulgaris* was more efficient at removing Cr (91%) and Mn (92%). The heavy metal removal efficiencies of our microalga and cyanobacterium are found to be higher than the efficiencies observed in previous studies with *C. vulgaris* and *A. variabilis* [36,43]. Although experimental study to unveil the heavy metal removal mechanisms of our microalgal and cyanobacterial species was not performed, we assume that our *C. vulgaris* and *A. variabilis* strains remove the heavy metals through biosorption, as a similar process has also been reported by previous studies regarding these microalgal and cyanobacterial species removing heavy metals (Fe^2+^, Mn^2+^, Zn^2+^, and Cd^2+^) from aqueous solution [60,61,62]. Both living and dead cells engage in the biosorption process, while heavy metal ions adhere to the functional groups on the cell surface and in the cytoplasm via various mechanisms including ion exchange, coordination or complexation, chelation, and micro-precipitation [63,64].

## 5. Conclusions

This study evaluated the potential of *C. vulgaris* and *A. variabilis* for the phycoremediation of raw polluted river water. The findings demonstrated that both microalgal and cyanobacterial species could be a great biological strategy for phycoremediation applications. Under the given experimental conditions, *A. variabilis* and *C. vulgaris* showed excellent efficiency in eradicating significant amounts of pollutants from the polluted water samples. The substantial growth of both *A. variabilis* and *C. vulgaris* was observed in polluted water samples, which demonstrated the ability of both microalga and cyanobacterium to withstand the environmental conditions of the polluted river water. The higher efficiency of *A. variabilis* in reducing EC, BOD, TDS, SO_4_^2−^, and Zn concentrations clearly suggests it is better suited than *C. vulgaris* for remediating those specific pollutants. However, *C. vulgaris* would be a more effective choice over *A. variabilis* for reducing water hardness (Ca^2+^ and Mg^2+^) as well Cr and Mn pollution. Nevertheless, the assessment of polluted water composition should be completed prior to the designing and application of microalgae- or cyanobacteria-based remediation technology, since the pollutant removal efficiency is found to be essentially species dependent.

## Figures and Tables

**Table 1:** Physicochemical properties of the raw water samples collected from Dhaleswari river, Bangladesh.

Parameters	Observed Values (*n* = 5)	Bangladesh Standards
pH	6.97 ± 0.12	6.5–8.5 [38]
EC (µS/cm)	1573.93 ± 32.79	800–1000 [39]
TDS (mg/L)	935.55 ± 24.00	500 [39]
BOD (mg/L)	17.06 ± 1.35	6 or less [38]
Ca^2+^ (mg/L)	84.04 ± 3.14	123 as total hardness (sum of Ca^2+^ and Mg^2+^) [33]
Mg^2+^ (mg/L)	69.54 ± 3.55	
SO_4_^2−^ (mg/L)	117.62 ± 4.50	200 [2]
Zn (mg/L)	0.35 ± 0.04	5 [41]
Cr (mg/L)	0.81 ± 0.03	0.1 [41]
Mn (mg/L)	0.65 ± 0.04	0.05 [41]

**Table 2:** Physicochemical properties of the river water before and after treatment by *C. vulgaris* and *A. variabilis*. W-CV and W-AV represent the water treated by *C. vulgaris* and *A. variabilis*, respectively, and W-C represents the untreated water (abiotic control); + denotes % increase and – denotes % removal. Values denoted by different lowercase letters (^a^, ^b^, ^c^) indicate significant (*p* < 0.05) differences in the pollutant removal efficiencies resulting from the treatments.

Parameters	Before Phycoremediation	After 20 days of Phycoremediation (*n* = 5)		
		W-CV (% +/−)	W-AV (% +/−)	W-C (% +/−)
pH	6.97 ± 0.12	8.07 ± 0.17 (+15.78%) ^a^	8.28 ± 0.11 (+18.79%) ^b^	7.45 ± 0.11 (+6.89%) ^c^
EC (µS/cm)	1573.93 ± 32.79	1388.10 ± 35.79 (−11.81%) ^a^	1014.63 ± 45.80 (−35.54%) ^b^	1496.46 ± 26.45 (−4.92%) ^c^
TDS (mg/L)	935.55 ± 24.00	684.24 ± 65.07 (−26.86%) ^a^	633.70 ± 6.20 (−32.26%) ^b^	869.23 ± 28.74 (−7.09%) ^c^
BOD (mg/L)	17.06 ± 1.35	8.97 ± 0.68 (−47.42%) ^a^	7.24 ± 0.74 (−57.56%) ^b^	13.24 ± 1.39 (−22.39%) ^c^
Ca^2+^ (mg/L)	84.04 ± 3.14	31.11 ± 2.81 (−62.98%) ^a^	48.63 ± 2.23 (−42.13%) ^b^	79.09 ± 2.87 (−5.89%) ^c^
Mg^2+^ (mg/L)	69.54 ± 3.55	19.22 ± 2.43 (−72.36%) ^a^	25.40 ± 2.72 (−63.47%) ^b^	62.14 ± 5.04 (−10.64%) ^c^
SO_4_^2−^ (mg/L)	117.62 ± 4.50	55.04 ± 1.87 (−53.21%) ^a^	38.77 ± 2.68 (−67.04%) ^b^	107.30 ± 3.30 (−8.77%) ^c^
Zn (mg/L)	0.35 ± 0.04	0.10 ± 0.02 (−71.43%) ^a^	0.04 ± 0.02 (−88.57%) ^b^	0.30 ± 0.05 (−14.29%) ^c^
Cr (mg/L)	0.81 ± 0.03	0.07 ± 0.02 (−91.38%) ^a^	0.24 ± 0.03 (−70.37%) ^b^	0.76 ± 0.02 (−6.17%) ^c^
Mn (mg/L)	0.65 ± 0.04	0.05 ± 0.02 (−92.31%) ^a^	0.16 ± 0.03 (−75.38%) ^b^	0.60 ± 0.03 (−7.69%) ^c^

**Table 3:** Phycoremediation efficiency (%) of different types of wastewaters by various algal species reported in previous studies and their comparison with the results of this study.

Type of Polluted Waters	Microalgae/ Cyanobacteria	pH	EC	TDS	BOD	Ca^2+^	Mg^2+^	SO_4_^2−^	Zn	Cr	Mn	References
River water	*C. vulgaris*	15.78	11.81	26.86	47.42	62.98	72.36	53.21	71.43	91.38	92.31	This study
	*A. variabilis*	18.79	35.54	32.26	57.56	42.13	63.47	67.04	88.57	70.37	75.38	
River water	*Chlorella* sp.	21.60	34.50	9.94	7.32	39.73	20.93	8.74	-	-	-	[4]
River water	*Scenedesmus* sp.	14–20	32	32	27.83	70.26	33.93	43.61	-	-	-	[29]
Textile wastewater	*A. variabilis*	36.51	57.14	41.18	56.36	15.69	22.97	31.67	45.71	-	-	[36]
	*N. muscorum*	28.58	54.29	37.25	54.55	13.73	18.92	23.33	43.57	-	-	
Sewage water	*C. vulgaris*	-	-	33.47–68.42	83.17–90.63	75.12–98.10	58.46–84.23	-	14.94–64.96	21.74–66.46	100	[43]
	*C. salina*	-	-	24.68–42.17	87.01–90.75	64.94–96.20	4.20–58.75	-	15.16–28.52	5.13–30.59	89.94–93.71	
Tannery wastewater	*Scenedesmus* sp.	48.21	-	-	35	-	-	-	65–98	81.2–96	-	[44]

## Data Availability

The datasets used and/or analyzed in this study are available from the corresponding author on reasonable request.
